# Sensorimotor perturbation-induced cortical responses by a novel PES system: analysis of the N1 component in healthy adults and Parkinson's disease

**DOI:** 10.3389/fnhum.2025.1668367

**Published:** 2025-10-07

**Authors:** Christian L. Rathke, Victor C. A. Pimentel, Caroline Cunha do Espirito-Santo, Gabriel A. M. Vasiljevic, André Felipe Oliveira de Azevedo Dantas

**Affiliations:** ^1^Postgraduate Program in Neuroengineering, Edmond and Lily Safra International Institute of Neuroscience, Santos Dumont Institute, Macaíba, RN, Brazil; ^2^Department of Mechatronics, Federal Institute of Rio Grande do Norte, Parnamirim, RN, Brazil

**Keywords:** electroencephalography, neurorehabilitation, sensorimotor integration, non-invasive stimulation, perturbation-evoked potentials, Parkinson's disease, message queue telemetry transport (MQTT), motor control

## Abstract

Perturbation-evoked potentials (PEPs) have been widely used to investigate static and dynamic perturbations on postural and motor control through analysis of cortical responses. In this pilot study, we present an innovative approach using IoT-based Perturbatory Electrical Stimulation (PES) during treadmill walking to assess cortical responses in healthy adults (*N* = 6) and individuals with Parkinson's disease (*N* = 4), with a focus on the N1 component. This approach integrates PES and EEG systems through an Internet of Things (IoT) framework utilizing the MQTT protocol, enabling synchronized and wireless data acquisition during gait. The results indicated significant differences in N1 latency (*p* = 0.005), with the Parkinson's disease group presenting higher latencies in the N1 component (252.50 ± 32.62 ms) compared to the healthy adult group (175.50 ± 30.42 ms). Significant correlations were observed between N1 amplitude and participants' age (r = 0.669, *p* = 0.049) and between TUG performance and PES intensity (mA) (r = -0.697, *p* = 0.037). No significant correlations were found between N1 latency and PES intensity (mA), visible motor threshold (mA), or Epworth Sleepiness Scale. These findings contribute to a better understanding of how Parkinson's disease impacts cortical responses to sensorimotor perturbations during gait, particularly regarding sensory processing and motor feedback, and highlight the potential utility of the PES system in future studies in motor control.

## 1 Introduction

Throughout evolution, bipedal walking has provided us with fundamental advantages for survival in various terrains (DeSilva, 2021). Maintaining balance is a complex process mediated by multiple peripheral and central structures (Takakusaki, 2017; Duysens and Forner-Cordero, 2018).

Daily life often exposes individuals to unexpected mechanical and sensory challenges (Dietz et al., 1989). Therefore, understanding the mechanisms involved in motor control is extremely important, helping to characterize motor dysfunctions and increased risks of falls (Jalilpour and Müller-Putz, 2023; Rieger et al., 2024).

Parkinson's disease (PD) affects a large portion of the global population, being characterized by direct impairment of motor and sensory control (Takakusaki et al., 2023; Morris et al., 2024), consequently resulting in deficits that increase the risk of falls, reduce autonomy, and worsen quality of life, which represent one of the leading causes of morbidity (Tomii et al., 2025; Murueta-Goyena et al., 2025).

In the PD population, falls are related to motor errors in adapting to environmental challenges due to deficits in anticipatory and compensatory postural adjustments. The literature describes that motor deficits in PD compromise gait initiation (Hou et al., 2024) and also present slower and less effective compensatory responses to sudden disturbances, reducing the ability to recover balance (Schlenstedt et al., 2017; Nonnekes et al., 2019).

Furthermore, there are still gaps in understanding how dysfunctions in PD may be associated with balance impairment, including sleep deprivation and sensory and cortical processing deficits (Gui et al., 2024; Lanir-Azaria et al., 2024; Heß et al., 2023). Studies such as Umemura et al. (2021) demonstrated that chronic sleep deprivation can impact balance in young adults, reinforce the need to include, in motor control and cortical studies, metrics that assess sleep deprivation (Naghavi and Aliasin, 2024).

Cortical involvement in motor control has been discussed over the years (Jacobs and Horak, 2007). The Perturbation-Evoked Potentials (PEPs), which have been widely used as a tool to investigate balancing control and locomotion, allow the analysis of cortical responses to different types of static and dynamic perturbations (Varghese et al., 2017; Dietz et al., 1984; Dimitrov et al., 1996; Duckrow et al., 1999).

Varghese et al. (2017) carried out a comprehensive literature review, highlighting the importance of PEPs for studying and describing motor control. According to the literature evaluated, most studies agree on the fact that PEPs are composed of a small positive potential (P1) observed around 30 to 90 ms after of the perturbation, followed by a large negative potential (N1) around 90 to 160 ms, and further by positive (P2) and negative (N2) potentials between 200 and 400 ms after the event.

However, the N1 component is one of the most studied due to its role in sensory processing and compensatory postural responses (Adkin et al., 2006; Mochizuki et al., 2009). Studies indicate that the N1 amplitude and latency can be modulated by factors such as perturbation predictability, sensory modality involved, and the complexity of the required motor response (Staines et al., 2001; Marlin et al., 2014).

In PD, Payne et al. (2022) found a smaller N1 amplitude width associated with severity of PD motor symptoms, lower functionality and mobility, reduced confidence in maintaining balance, and lower overall cognitive function. In young adults, Payne and Ting (2020) reported a significant interaction between balancing performance and the effect of perturbation magnitude on cortical N1 amplitude, suggesting greater cortical activation to meet the demands of postural adjustments.

Furthermore, Mirdamadi et al. (2025) reported that the N1 component exhibits reduced amplitude and increased latency in older adults compared to younger individuals. Overall, the literature indicates that the N1 component is modulable and adaptive in young adults, yet demonstrates diminished amplitude in both aging and Parkinson's disease (PD). In PD, this reduction is strongly associated with clinical severity, whereas in older adults it remains unclear whether the observed alterations reflect a normative aging effect or a pathophysiological process analogous to that seen in the disease.

Different pathways can evoke PEPs, the tools mentioned in the literature commonly used for induced gait disturbances in balance and motor control studies are based on treadmills and robotic actuators (Brough and Neptune, 2024; Ciunelis et al., 2024; Chodkowska et al., 2024; Roeles et al., 2018), auditory systems (Forner-Cordero et al., 2014; Duppen et al., 2024; Cornwell et al., 2020), visual stimuli (Riem et al., 2020; Hao and Siu, 2021; Tran et al., 2023), subsensory stimulation (Bassiri et al., 2022), and electrical stimulation (Dietz et al., 1985; Nishimura et al., 2018; Kozasa et al., 2020; Kim et al., 2024).

Our system offers novel features related to wireless connectivity, mechanism of action, and mobility, providing the possibility of integration with EEG and other devices through an MQTT protocol. This enables approaches that offer increased flexibility for studies in open environments, eliminating the need for highly robust and costly laboratory systems.

In this pilot study, we aim to evaluate cortical responses to sensorimotor perturbations induced by a novel Perturbatory Electrical Stimulation (PES) system, comparing N1 latency responses in healthy adults and individuals with Parkinson's Disease. We hypothesize that individuals with PD will present altered N1 responses compared to adults without the condition, reflecting possible changes in somatosensory sensitivity and cortical processing.

## 2 Methods

### 2.1 Participants

The sample consisted of *N* = 10 subjects; the exclusion criteria were a history of musculoskeletal injuries in the lower limbs and discomfort or sensorimotor intolerance to electrical stimulation. Additionally, for the Parkinson's disease group, there are no deep-brain stimulation (DBS) or cardiac pacemaker implants. Participants were divided into two groups: the Healthy Adult Group (GA), *N* = 6, aged between 19 and 41 years, and the Parkinson's Group (PD), *N* = 4, which included individuals aged between 48 and 58 years.

### 2.2 Medications status and other substances

All participants were instructed not to consume any stimulant or alcoholic beverages for 12 h before the protocol. Participants with PD were asked to continue taking their usual medications, including Levodopa (L-DOPA), at least one hour before the protocol.

### 2.3 Sleep and functionality assessment

Sleep quality and functionality were assessed in all participants prior to the protocol. To evaluate sleep, we used the Epworth Sleepiness Scale questionnaire (Gonçalves et al., 2023). The functionality assessment involved the 3-meter TUG Test, widely used to assess fall risk in the elderly population (Zhou et al., 2025).

### 2.4 IoT PES system

The device used was previously developed by De Almeida et al. (2022), and has been used in different studies. Bertucci et al. (2022); Henrique e Silva Bezerra et al. (2022); Leal et al. (2023); Gonzalez-Cely et al. (2024), due to its practicality and adaptability to personal software tailored to meet researchers' needs.

Our codes are stored in a library named *Neurodevices.bib*, available on GitHub. The Hardware consists of a portable electrical stimulator managed by a microcontroller, capable of generating multiple types of current, including functional electrical stimulation (FES), along with the capacity to communicate with other devices via the MQTT protocol.

### 2.5 Mechanism of gait perturbation and rectus femoris muscle

The gait cycle is defined as a cyclical pattern that occurs during walking and begins when the heel of one foot touches the ground and ends when the same heel touches the ground again (Perry and Burnfield, 2024). Balance is ensured through the orchestration of the upper limbs, trunk, and lower limbs; consequently, the body can become unbalanced, affecting different body segments or muscles involved in motor control.

In our initial study, we chose the rectus femoris muscle of the volunteer's dominant limb for PES application. The rectus femoris muscle is primarily responsible for knee extension and hip flexion. It also plays a role in stabilizing the knee and supporting other muscles during the initial and final phases of the cycle. Its most evident role is in the pre-swing phase, where it acts as a hip flexor, helping to initiate the forward movement of the leg (Neumann, 2010).

Our objective was to generate involuntary contractions through electrical perturbations across all phases of the gait cycle. Nevertheless, we did not have control over the exact phases in which the stimuli occurred; we hypothesized that the most evident ones would be present when applied in the pre-swing phase, forcing excessive knee extension and a motor error similar to tripping over an obstacle.

### 2.6 Perturbation stimuli

The frequency of electrical stimuli was set at 60 Hz and a pulse width of 200 μ*s* for all participants. These parameters were based on the literature of functional electrical stimulation (FES), where the frequency typically ranges from 50 to 150 Hz, while the pulse duration can range from 60 to 400 μ*s* (Aout et al., 2023).

The PES system is capable of generating stimuli at random times, and was programmed to trigger between 30 and 60 second intervals, making all disturbances unpredictable and ensuring an average of 15 or 17 disturbances during the protocol (12 min). The stimulation generated induces an involuntary muscle contraction, purposefully disturbing the gait cycle at different percentages, forcing the individual to respond with compensatory adjustments of different intensities depending on the gait phase where the stimulus was applied.

**Figure 1:**
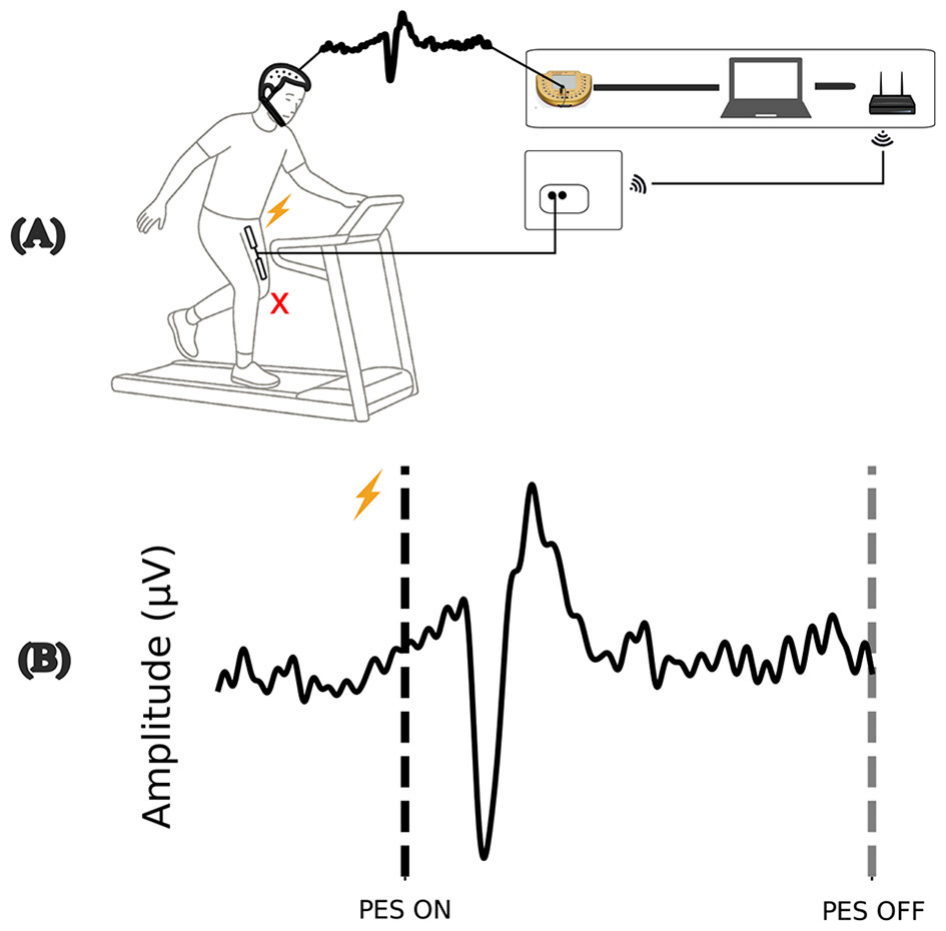
Illustration of the perturbation, **(A)** involuntary muscle contraction promotes a disturbance during the gait cycle. **(B)** Example of the evoked cortical response during perturbation by PES ON. Source: Author.

The characteristics of the perturbation are illustrated in Figure 1. After the contraction, the individual is forced to make adjustments to maintain a balanced center of mass. One of the most common strategies used in response to stumbles or motor planning errors is adjusting step width.

### 2.7 Electroencephalography setup

The cortical signals were captured with a V-amp amplifier with 16-channel electrodes at a sampling frequency of 512 Hz. The impedance was measured ensuring values lower than < 10*kΩ*, and the electrodes were positioned in Fp1, Fp2, F3, F4, FC1, FCz, FC2, C3, C1, Cz, C2, C4, CP1, CPz, CP2, and Pz sites based on 10–20 system (Herwig et al., 2003). Furthermore, the analyses were conducted in the channel (Cz), a key region for the study of the powers evoked by disturbances in previous studies (Varghese et al., 2017).

The OpenViBE acquisition software was used and integrated with the PES system to manage EEG signal acquisition. This setup allows for the synchronous capture of cortical activity with the stimuli generated by the PES system.

### 2.8 Protocol of gait perturbations

Initially, all volunteers passed a pre-protocol, in which the intensity of electrical stimulation was determined based on tolerance (discomfort) and visible contraction of the rectus femoris muscle (dominant limb) using self-adhesive electrodes fixed at a distance of 15 cm between electrodes in the central portion of the rectus femoris muscle. After adjusting the required stimulation parameters and EEG clothing, volunteers were positioned on the treadmill and instructed to select a comfortable walking speed.

**Figure 2:**
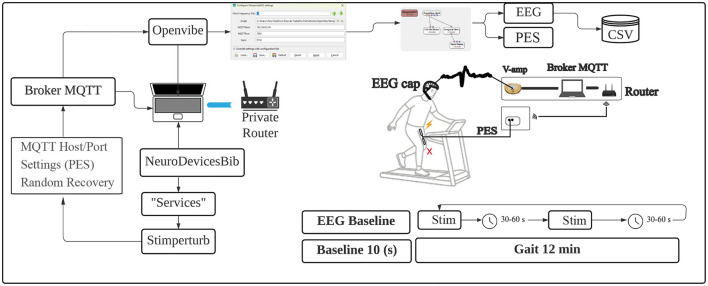
Protocol framework illustration. Source: Author.

The Figure 2 illustrates the protocol framework; the first 10 seconds of walking were recorded as a baseline for EEG recording, and after the baseline, the first perturbation was triggered as programmed, for 1,000 (ms). The active rest period (30–60s) was randomly selected, and the next stimulus occurred only after the defined time had elapsed, with all perturbations being unpredictable over time.

The total protocol time was 12 min of continuous walking without interruptions, and on average, 15 to 17 perturbations were generated, with the aim of avoiding excessive fatigue for the volunteers. Data collection was completed, and two files were generated: one containing the EEG data and another with the perturbation time records, both generated by OpenViBE and saved in CSV format for later analysis.

### 2.9 Data processing

#### 2.9.1 Electroencephalography data processing

**Figure 3:**
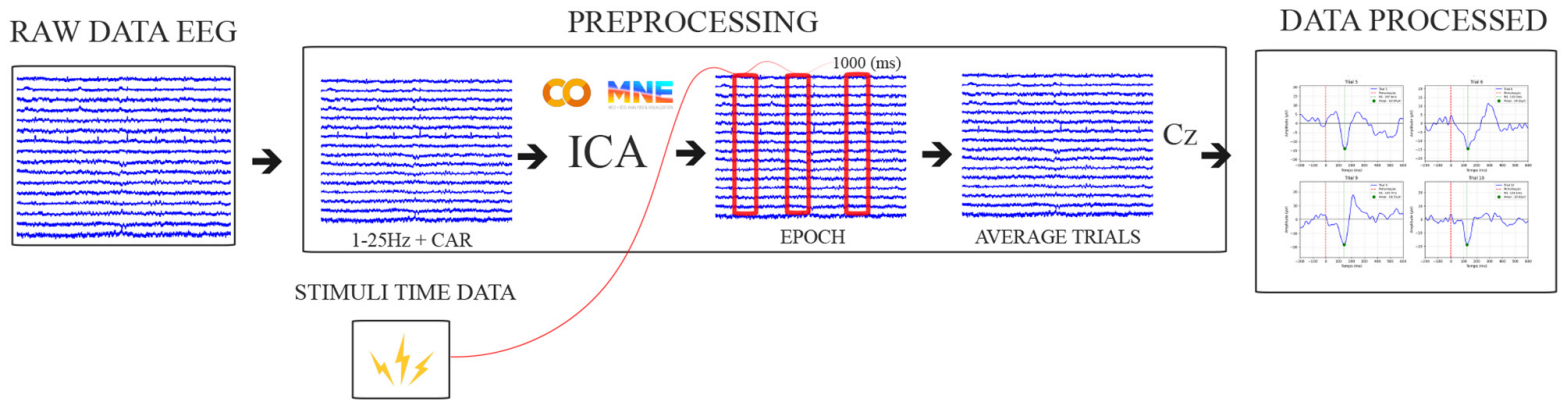
Pipeline data processing illustration. Source: Author.

The data processing is illustrated in Figure 3, based on a literature review (Luck, 2014; Varghese et al., 2017), with a focus on removing low-frequency noise, attenuating frequencies above 25 Hz. First, a high-pass filter at 0.1 Hz was applied to attenuate DC and low-frequency noise in raw EEG data, and a band-pass filter of 1–25 Hz was used, followed by a CAR filter to remove common noise.

Additionally, the network artifacts and noise were removed using the Independent Component Analysis (ICA) algorithm available in the MNE-Python (Gramfort et al., 2013). Signals from the Cz electrode were then baseline-corrected by subtracting the average signal within the 500 (ms) preceding the perturbation event, and the signals were subsequently segmented into 1,000 ms epochs, starting 500 ms before the perturbation onset.

The cortical responses were averaged from perturbation events per participant across trials. The cortical N1 latencies (ms) were identified at the negative peak between 100 and 300 ms after perturbation onset.

### 2.10 Statistical analysis

Data normality was assessed using the Shapiro-Wilk test, and homogeneity of variances was evaluated using the Brown-Forsythe test. Most variables followed a normal distribution (*p*>0.05) and exhibited homogeneous variances. Exceptions were found in the Epworth Sleepiness Scale scores in the GA group and treadmill walking speed in both groups, which violated normality assumptions.

For between-group comparisons, Student's *t-*test was used for normally distributed variables, while the Mann-Whitney U test was applied for non-parametric data. To enhance robustness in continuous signal analysis, a point-wise two-sample *t*-test comparison of Perturbation-Evoked Potentials (PEP) was conducted using the SPM1D package (Pataky, 2012) for Python, with full results available in the Supplementary material.

Correlation analyses were performed using Pearson's correlation coefficient for normally distributed data. All statistical analyses were executed in JASP (version 0.18.1.0) and Python (version 3.9).

## 3 Results

Table 1 presents the clinical characteristics of the participants in the study, divided into two groups: the healthy adults group (GA) and the individuals with Parkinson's disease (PD). The GA group consisted of six participants (5 males and 1 female) with a mean age of 28.33 ± 7.74 years. They had a mean height of 174 ± 12.72 cm, mean weight of 78.5 ± 20.88 kg, and a mean Epworth Sleepiness Scale of 7.66 ± 4.22. The mean performance time in the Timed Up and Go (TUG) test for this group was 7.22 ± 1.11 seconds.

**Table 1:** Demographic, clinical, and experimental measures for GA and PD groups with statistical comparisons.

**Variables**	**GA (mean ±SD)**	**PD (mean ±SD)**	**p**	**Effect size**
**Demographics**				
Age (y)	28.33 ± 7.74	53.50 ± 4.20	< 0.001^**^	–3.79
Height (cm)	174 ± 12.72	167.25 ± 10.07	0.402	0.57
Weight (kg)	78.5 ± 23.20	80.92 ± 12.24	0.854	–0.12
Sex (M/F)	5M/1F	3M/1F	–	–
**Clinical**				
Time Up And Go (s)	7.22 ± 1.11	8.30 ± 1.71	0.259	–0.79
Hoehn and Yahr	–	1.62 ± 0.47	–	–
Years of diagnosis (y)	–	1.1 ± 0.66	–	–
Medication status	–	ON	–	–
Epworth scale	7.66 ± 4.22	12.50 ± 5.06	0.128	–0.63
**Experimental**				
Vis. motor thresh. (mA)	8.66 ± 4.32	16.50 ± 5.26	0.032^*^	–1.67
Intensity PES (mA)	19.3 ± 8.45	30.50 ± 9.29	0.084	–1.27
Self-select velocity (km/h)	3.25 ± 0.27	2.35 ± 0.70	0.060	0.75
N1 latency (ms)	175.50 ± 30.44	252.50 ± 32.62	0.005^**^	–2.46
Amplitude (μV)	–5.33 ± 4.46	–3.45 ± 3.58	0.501	–0.46

Independent samples *t*-test: student's t (parametric), Mann-Whitney U (non-parametric). Effect sizes: Cohen's d (parametric), rank-biserial correlation (non-parametric). ^*^p < 0.05; ^**^p < 0.01.

The PD group included four participants (3 males and 1 female) with a mean age of 53.50 ± 4.20 years. They presented a mean height of 167.25 ± 10.71 cm and a mean weight of 80.92 ± 12.24 kg. The mean duration of Parkinson's disease diagnosis was 1.1 ± 0.66 years, with an average Hoehn and Yahr stage of 1.62 ± 0.47, and the mean Epworth Sleepiness Scale score was 12.50 ± 5.06. The mean Time Up And Go test time was 8.30 ± 1.71 seconds.

**Figure 4:**
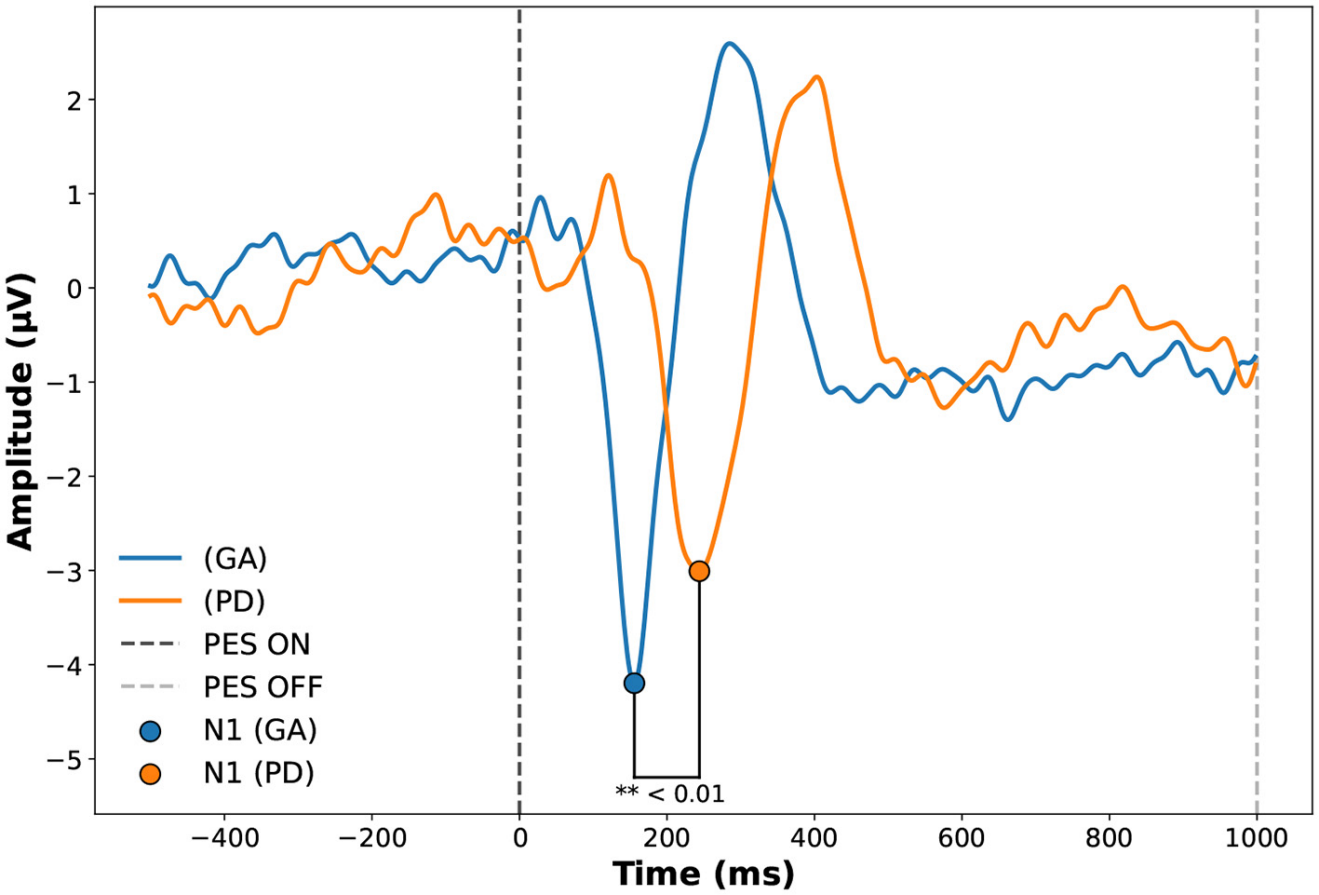
Evoked potential in Cz of the Adult Healthy Group (GA) and Parkinson (PD) groups during Perturbatory Electrical Stimulation (PES). The figure shows the average PEP responses of participants before, during, and after PES. The vertical line at 0 ms indicates PES onset (PES ON), while the line at 1,000 ms marks the end of stimulation (PES OFF). The comparison highlights group-specific differences in the evoked potentials during the stimulation. Source: Author.

Group comparisons were performed using Student's t-test for normally distributed data and the Mann-Whitney U test for non-normally distributed data, with the null hypothesis stating no differences between groups, as illustrated in Table 1. N1 latency (ms) and Motor Threshold showed significant differences between groups (*p* = 0.005) and (*p* = 0.032). However, N1 Amplitude, TUG test, PES Intensity, and Epworth Sleepiness Scale did not show significant differences (*p* = 0.501), (*p* = 0.259), (*p* = 0.084), and (*p* = 0.128) respectively. Disturbances generated by the system are described through the analysis of the evoked potential around the events, illustrated by the average of PEPs between the groups in Figure 4.

The PD group presented higher response latencies in the N1 component, as shown in Table 2. The healthy adult group (GA) had a mean of 175.50 ± 30.44 (ms), while the Parkinson's disease group (PD) had a mean of 252.50 ± 32.62 (ms). We observed a strong correlation between evoked potential amplitude (μV) and participant age (*r* = 0.669, *p* = 0.049 for Pearson), and TUG with PES intensity (*r* = –0.697, *p* = 0.037 for Pearson).

**Table 2:** Individual's average trials latency and amplitude of N1 component.

**ID**	**GA 1**	**GA 2**	**GA 3**	**GA 4**	**GA 5**	**GA 6**	**PD 1**	**PD 2**	**PD 3**	**PD 4**
N1 latency (ms)	199	177	150	138	220	169	297	253	220	240
N1 amplitude (μV)	–2.4	–5.53	–6.44	–13.43	–0.8	–3.41	–1.28	–8.4	–3.7	–0.40
Trials	16	16	17	15	16	16	15	16	15	17

Additionally, no significant correlation was found between N1 amplitude and PES intensity (*r* = 0.344, *p* = 0.365 for Pearson), or between N1 Latency and PES intensity, respectively (*r* = –0.030, *p* = 0.940 for Pearson). Other non-significant correlations included latency N1 with amplitude (*r* = 0.565, *p* = 0.113 for Pearson) and Epworth scale (*r* = 0.574, *p* = 0.106 for Pearson), not reaching the conventional statistical significance limit of *p* < 0.05.

## 4 Discussion

The analysis of latency in the N1 component showed a significant difference between the groups, with the PD group presenting higher latencies 252.50 ± 32.624 (ms) compared to the young adult group GA 175.50 ± 30.44 (ms), as illustrated in Figure 4. Previous studies show that the delay in cortical response observed in the PD group may be attributed to alterations in central motor and somatosensory pathways typical of Parkinson's disease, which undergo structural degradation and cortical processing overload (Takakusaki et al., 2023; Boebinger et al., 2024).

Chu et al. (2024) describes that basal ganglia hyperactivity generates excessive inhibition of the thalamus, impairing transmission to the primary motor cortex (M1). Additionally, abnormal synchronization of beta oscillations (13–30 Hz) compromises neural flexibility. The loss of glutamatergic innervation and reduced synaptic plasticity, associated with decreased dopamine levels, further slows motor control in Parkinson's disease. Together, these factors may contribute to the observed increase in N1 latency.

The literature shows that increased N1 latency is not exclusive to Parkinson's disease. Mirdamadi et al. (2025) compared young and elderly adults and attributed the increase in latency to natural aging, likely due to changes in cortical excitability and slower neural processing. In our PD sample, latency was approximately 22% higher than the values reported by these authors for older healthy adults (206.6 ± 35.3 ms), despite our sample having a younger mean age of 58 years compared to 70 years in their study.

Additionally, our healthy adult group showed a 10.4% increase in latency (175.50 ± 30.44 ms) compared to the authors' reported values (159 ± 14.4 ms).This may suggest that, although aging influences some of our findings, the presence of the disease or other additional factors, such as more complex tasks during protocol, may be contributing to the increased N1 latency.

In a study involving static platform disturbances (Payne et al., 2022), reported mean N1 latencies below 200 ms, with no significant difference between elderly individuals with and without PD. The mean ages of the Parkinson's group and the control group were 71 years and 69 years, respectively, both higher than the mean age of our sample. However, we still observed higher latencies, which reinforces the hypothesis that the delay in N1 latency in our sample could not be attributed solely to natural aging, and that pathological or functional factors related to balance and cognition may be more strongly involved.

We agree that individual variability in N1 characteristics may reflect differences in balance and cognitive function, rather than being determined exclusively by the pathological effects of PD. Indeed, within the PD group in the study by Payne et al. (2022), earlier and narrower N1 peaks with higher amplitudes were associated with greater severity of motor symptoms, lower balance confidence, and slower gait speed, indicating that specific temporal characteristics of the N1 may function as indirect predictors of functional impairment.

These results reinforce the idea that aging and neurodegenerative conditions may influence N1 latency through different, possibly interacting, mechanisms, and that a more detailed analysis of individual-level associations may provide more insights than group-level comparisons. In other study (Payne and Ting, 2020) suggests that N1 amplitude may also reflect balance health in young adults and observed that higher N1 amplitudes were associated with greater difficulty in recovering balance, especially in individuals with lower functional capacity or exposed to more intense perturbations, suggesting greater cortical involvement.

Complementarily, Mirdamadi et al. (2025) demonstrated that the N1 amplitude has excellent test-retest reliability (ICC > 0.9) and can be considered a reliable parameter even with a small number of trials. Furthermore, they found that older adults had significantly lower amplitudes (26.4 ± 9.8μV) than young adults (44.2 ± 19.3 μV), in addition to the aforementioned longer latency.

Although in our study, the N1 amplitude did not differ significantly between groups (GA: –5.33 ± 4.46 μV; PD: –3.45 ± 3.58 μV), its low magnitude suggests the perturbations may not have been sufficiently challenging to fully engage cortical responses. The values were significantly lower than those reported in the literature (Varghese et al., 2017).

Individual variability in muscle recruitment, task familiarity, and preparation strategies may also have influenced the magnitude of cortical responses. Future studies could explore higher-intensity stimulation, alternative muscle groups, or preparatory tasks to elicit more pronounced cortical activity. These adjustments may improve the effectiveness of the generated disturbances, making them more challenging.

We observed that subjects in the PD group required higher average stimulation intensities to reach their maximum tolerance (discomfort) to the PES, highlighting the role of sensory processing alterations in postural and gait deficits in Parkinson's disease (PD).

According Takakusaki et al. (2023), sensory integration is impaired at both subcortical and cortical levels due to insufficient cholinergic and monoaminergic neurotransmitters, affecting the thalamus and primary temporoparietal cortex. This disruption compromises proprioception, vestibular, and visual perception, contributing to postural instability and deformities. These findings suggest that altered proprioception and multisensory processing may impair central nervous system responses, ultimately affecting postural control and gait.

Classic studies by (Dietz et al. 1984, 1985, 1989) demonstrate that the amplitude of N1 ranges from 0.8 to 80 μV, depending on various factors manipulated in the experiments. During gait, there is a suppression of cortical evoked potentials, resulting in reduced amplitudes and greater latencies compared to static posture conditions. However, according to the author, walking also increases the latency of evoked potentials and the combination of the effect of the disease and the motor context may suggest the high latency values found in our study.

In addition to the neurophysiological aspects and the static or dynamic condition of the disturbance, the cortical response to a disturbance is directly related to the emotional elements and the individual strategies adopted by each subject in response to the disturbance (Palmer et al., 2021).

The correlations between N1 amplitude and PES intensity (mA) (r = 0.344, *p*=0.365), as well as between PES latency and intensity (mA) (r = –0.030, *p*=0.940), corroborate the fact that electrical stimulation is not directly correlated with signal generation through noise, which is an important finding that contributes to the validation of our system, and it is also possible to observe that the stimulus lasted 1,000 (ms). The Evoked N1 component was concentrated around 100 to 300 (ms), as shown in Figure 4.

Regarding secondary aspects, although not statistically significant, the PD group presented a higher overall average score on the Epworth Sleepiness Scale (12.50) compared to the GA group (7.66), suggesting a tendency for individuals with Parkinson's to be more prone to episodes of daytime sleepiness. This finding is consistent with the literature, as GABAergic neurotransmission is essential for regulating sleep, and the alterations caused by PD contribute to poor sleep quality and increased daytime sleepiness. Despite significant age differences between groups, functional performance, as measured by the Timed Up and Go (TUG) test, was similar (GA: 7.22 ± 1.11 s; PD: 8.30 ± 1.71 s), indicating that basic mobility was preserved across groups.

In summary, our findings indicate that N1 latency is significantly prolonged in individuals with Parkinson's disease compared to healthy adults, while N1 amplitude remains similar. Correlations with age and functional measures highlight the complex interplay between disease, aging, and motor context, reinforcing the relevance of cortical evoked potentials as markers of postural control and functional integrity.

We also emphasize the innovative nature of our perturbation system and its potential for future research. However, multiple factors may influence the observed differences in latencies and amplitudes between groups, so these findings should be interpreted with caution. Future studies with larger sample sizes, age-matched controls, OFF-medication assessments, and precise control of the gait cycle phase during perturbations will be essential to confirm and extend our observations, as well as to isolate effects specifically related to Parkinson's disease.

## 5 Conclusion

The results indicate that N1 latency is significantly prolonged in individuals with PD, while amplitude remains similar, suggesting delayed cortical processing in motor control. Despite higher Epworth Sleepiness Scale scores in PD, highlighting the complex interplay between aging, sensory processing, and motor function.

Our IoT PES + EEG system, one of the first to generate perturbations in a fully integrated and systematic manner during gait in PD, successfully evoked cortical potentials, demonstrating its innovative potential for probing somatosensory and motor pathways. These findings open avenues for future studies integrating complementary devices, such as virtual reality, accelerometers, or instrumented insoles, to improve evaluation and rehabilitation strategies in PD.

## Data Availability

The raw data supporting the conclusions of this article will be made available by the authors, without undue reservation.
