# Supplementary figures and images for: Sensorimotor perturbation-induced cortical responses by a novel PES system: analysis of the N1 component in healthy adults and Parkinson's disease
Source: Front Hum Neurosci. 2025 Oct 7;19:1668367. doi: 10.3389/fnhum.2025.1668367 (PMC12537702; doi:10.3389/fnhum.2025.1668367)

**A)**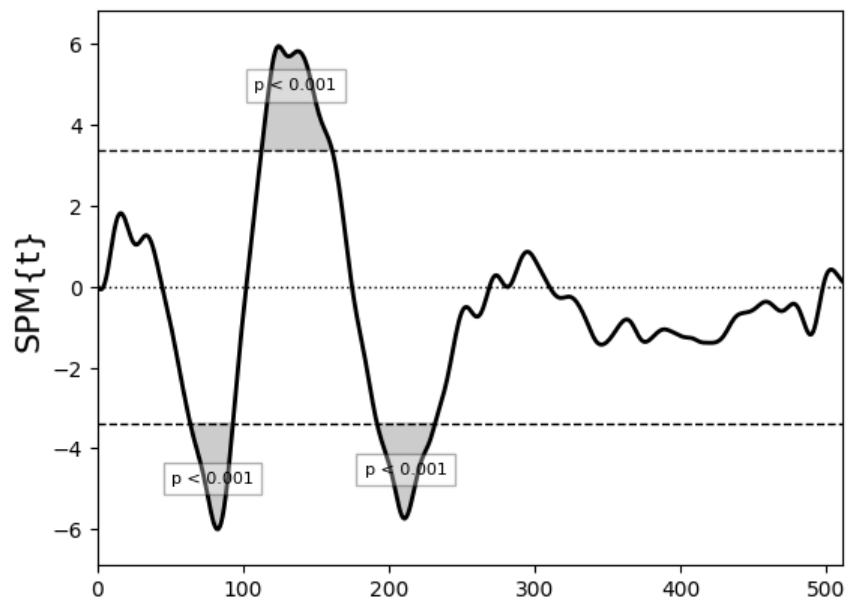**B)**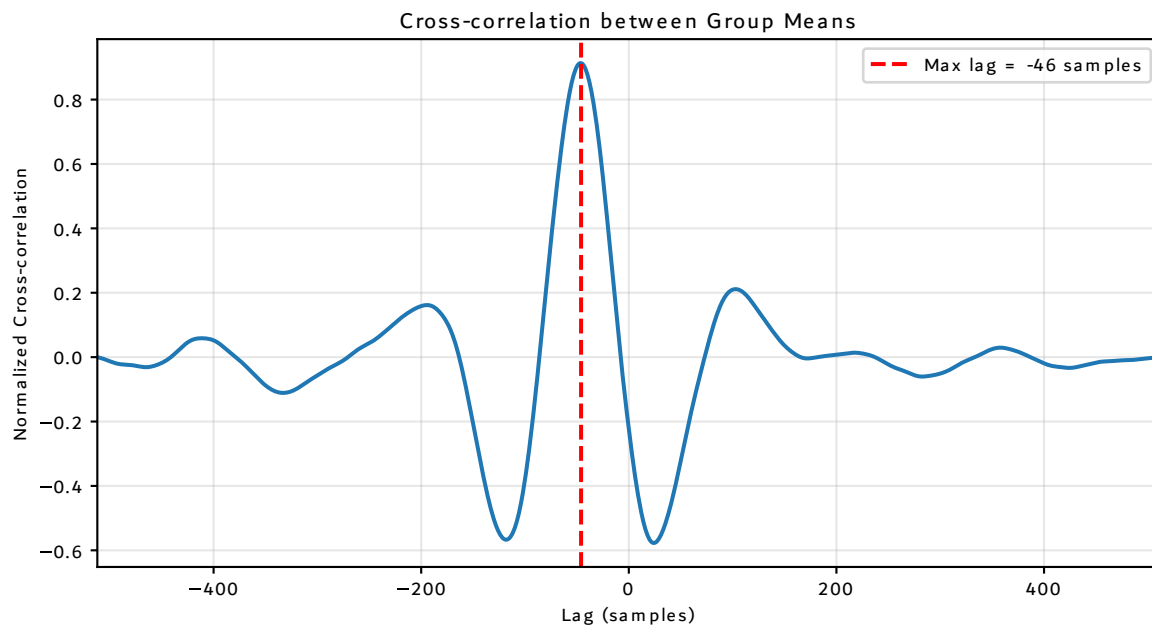

Supplement: Supplementary file 1 [file Supplementary_file_1.zip › Supplementary Material/Data Sheet 1.PDF]

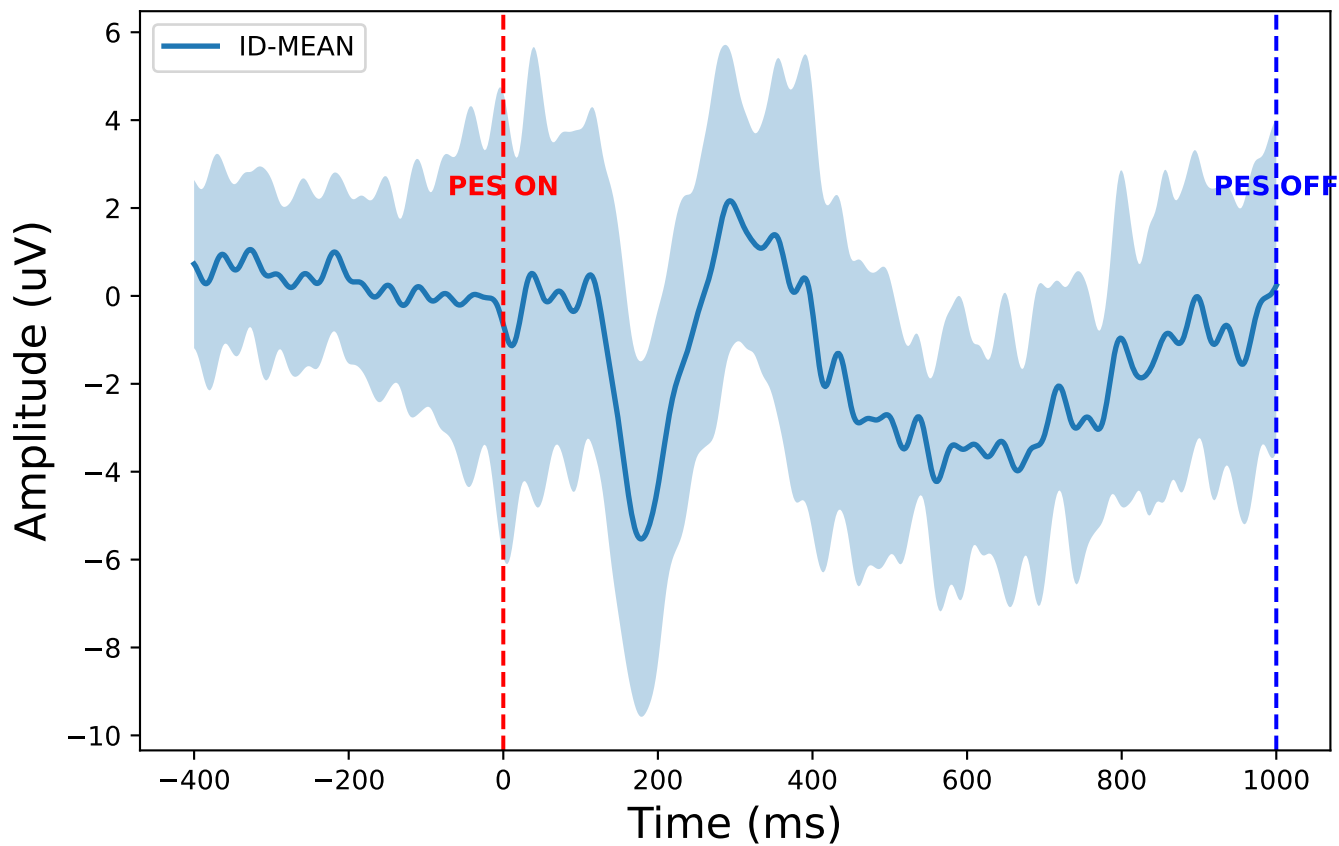

Supplement: Supplementary file 1 [file Supplementary_file_1.zip › Supplementary Material/Data Sheet 10.PDF]

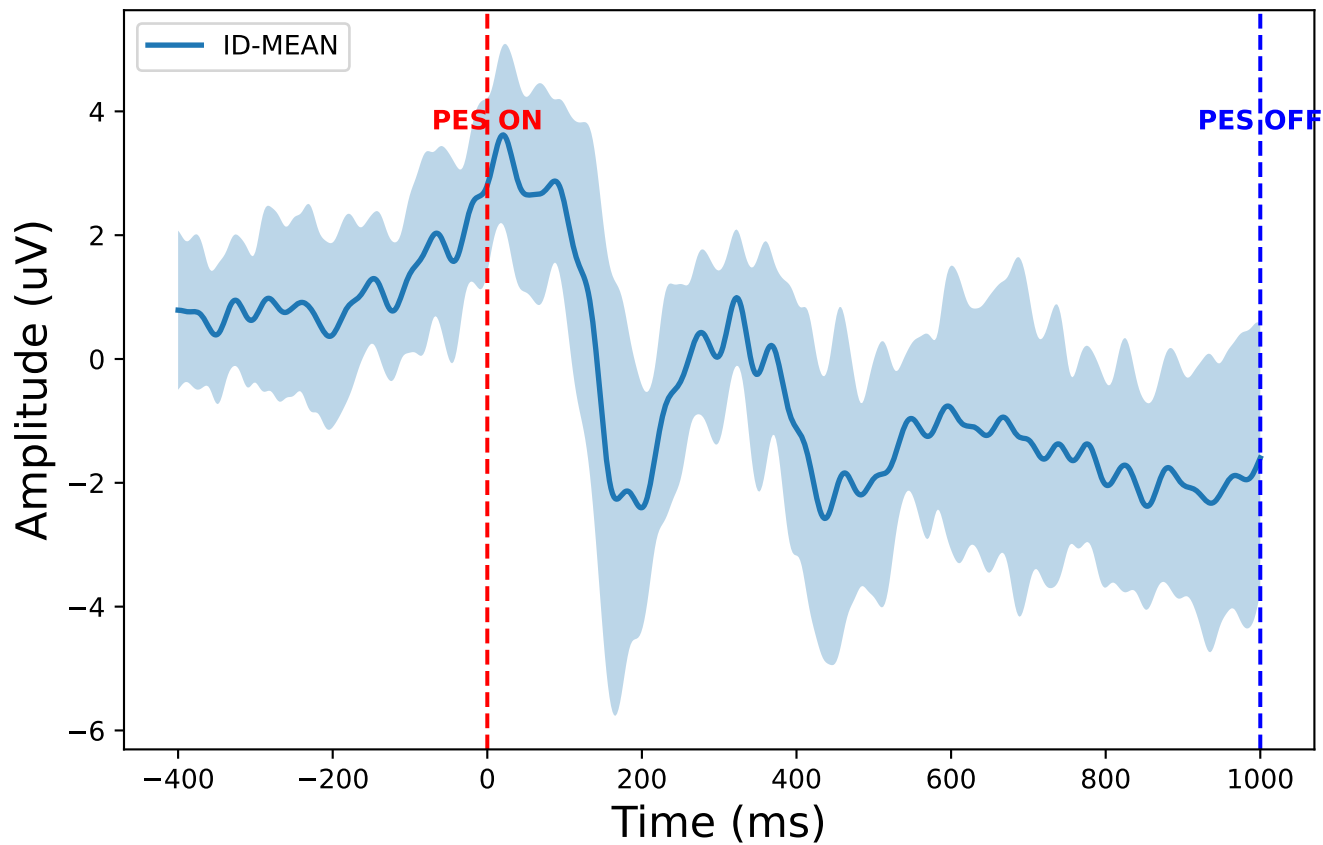

Supplement: Supplementary file 1 [file Supplementary_file_1.zip › Supplementary Material/Data Sheet 11.PDF]

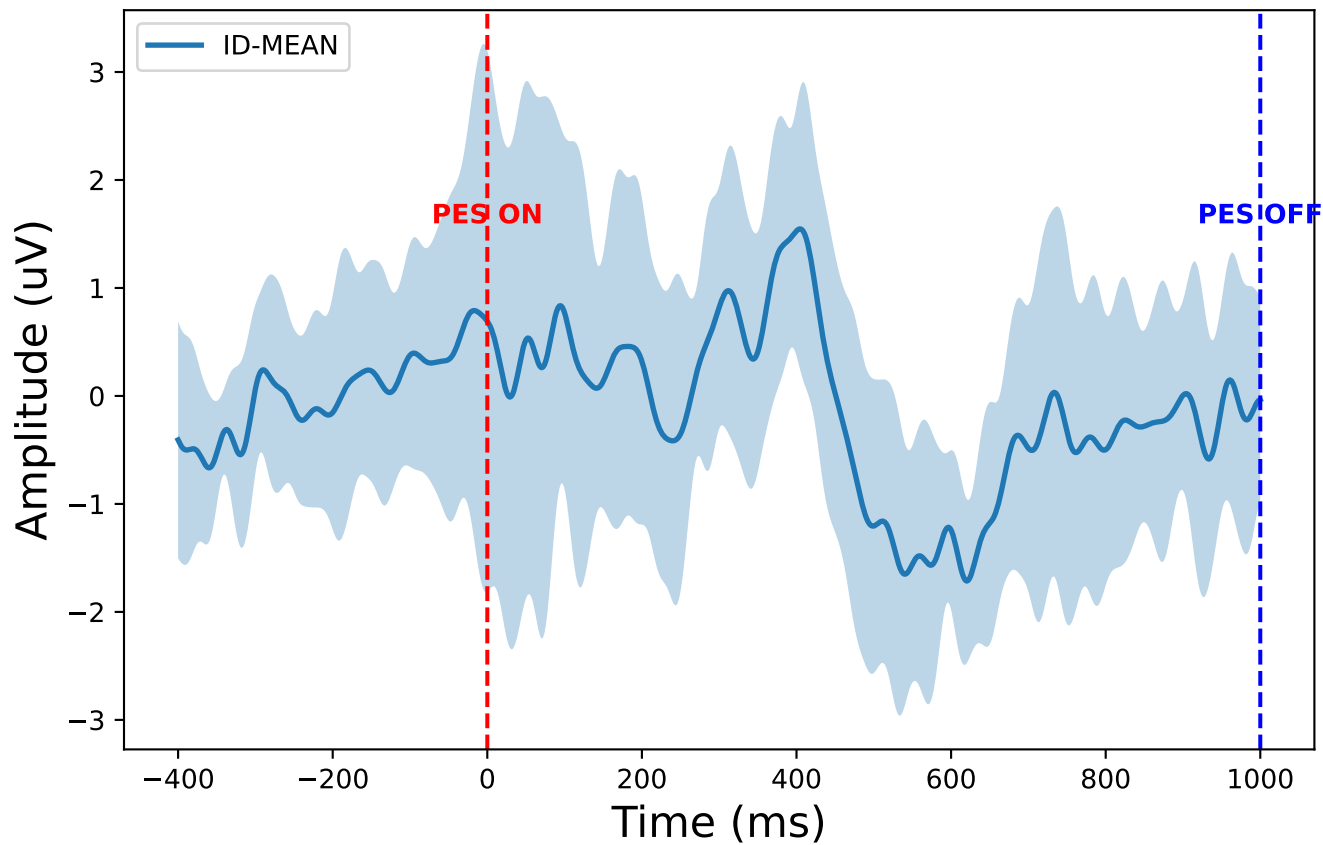

Supplement: Supplementary file 1 [file Supplementary_file_1.zip › Supplementary Material/Data Sheet 2.PDF]

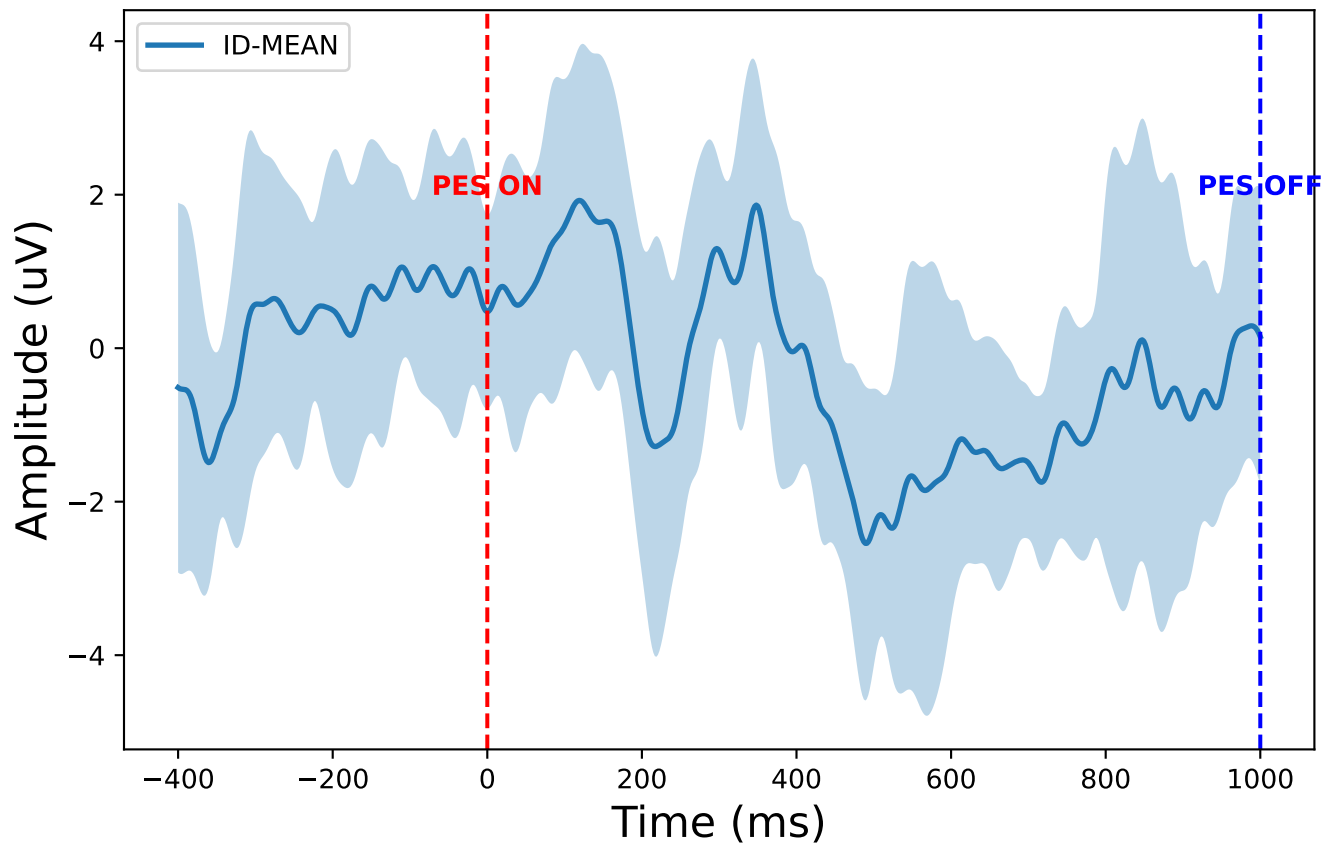

Supplement: Supplementary file 1 [file Supplementary_file_1.zip › Supplementary Material/Data Sheet 3.PDF]

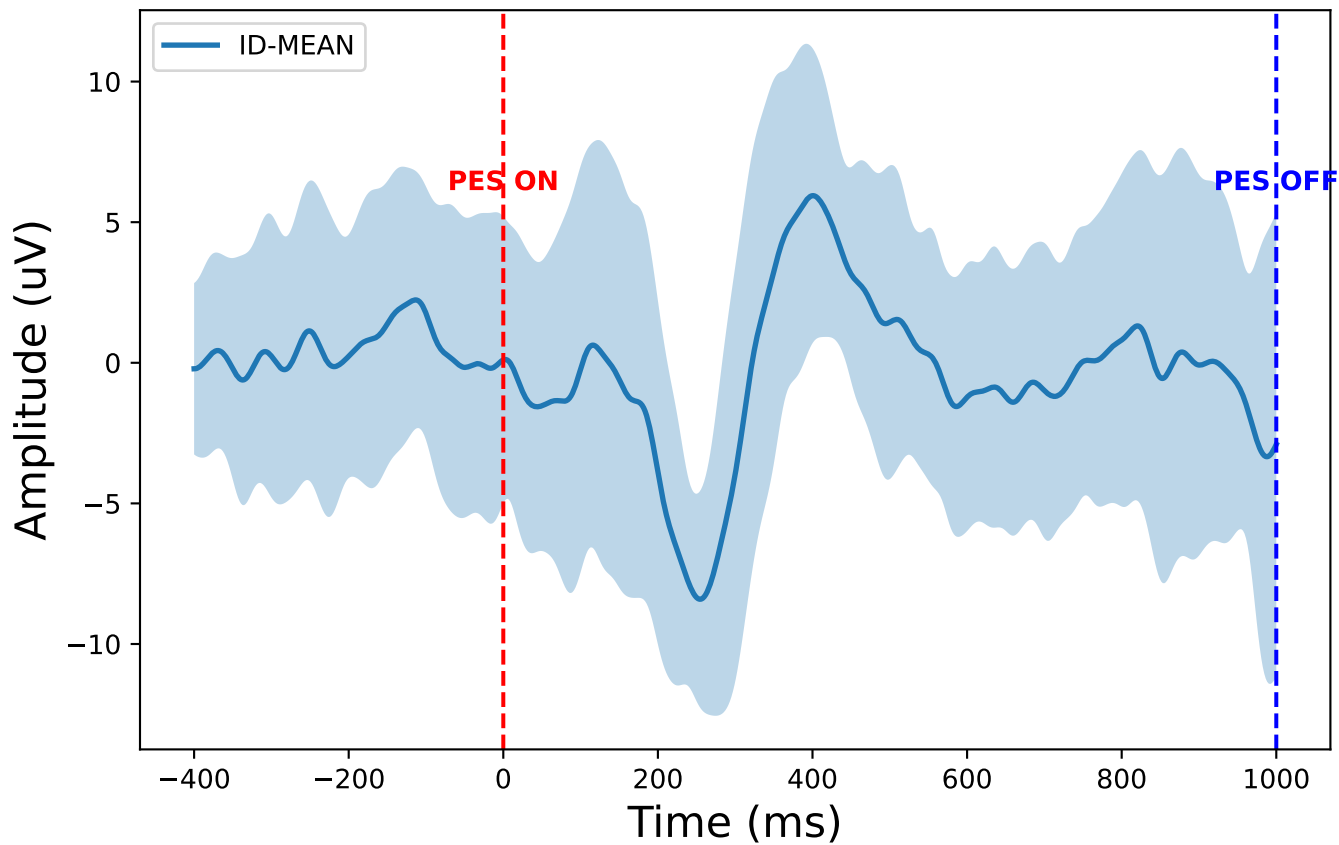

Supplement: Supplementary file 1 [file Supplementary_file_1.zip › Supplementary Material/Data Sheet 4.PDF]

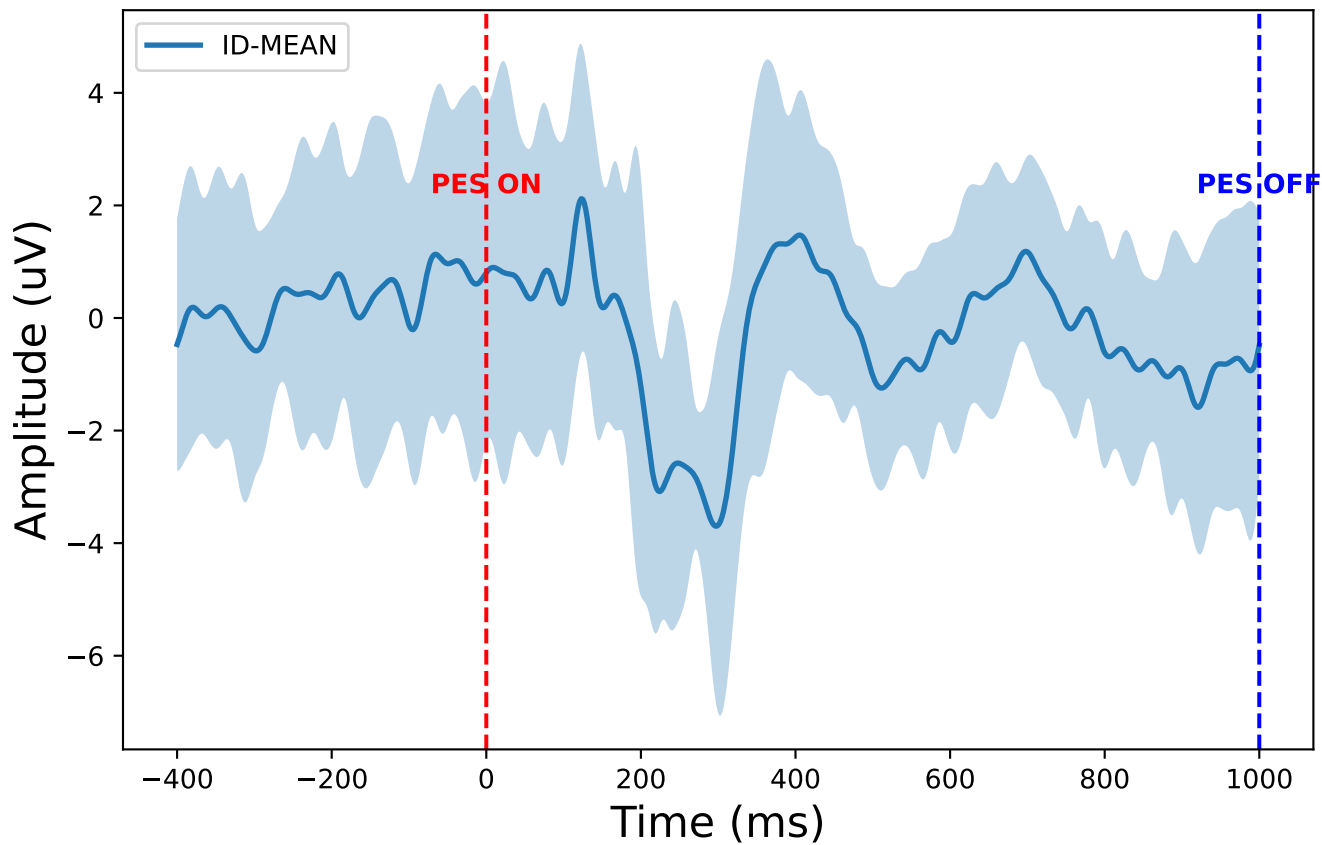

Supplement: Supplementary file 1 [file Supplementary_file_1.zip › Supplementary Material/Data Sheet 5.PDF]

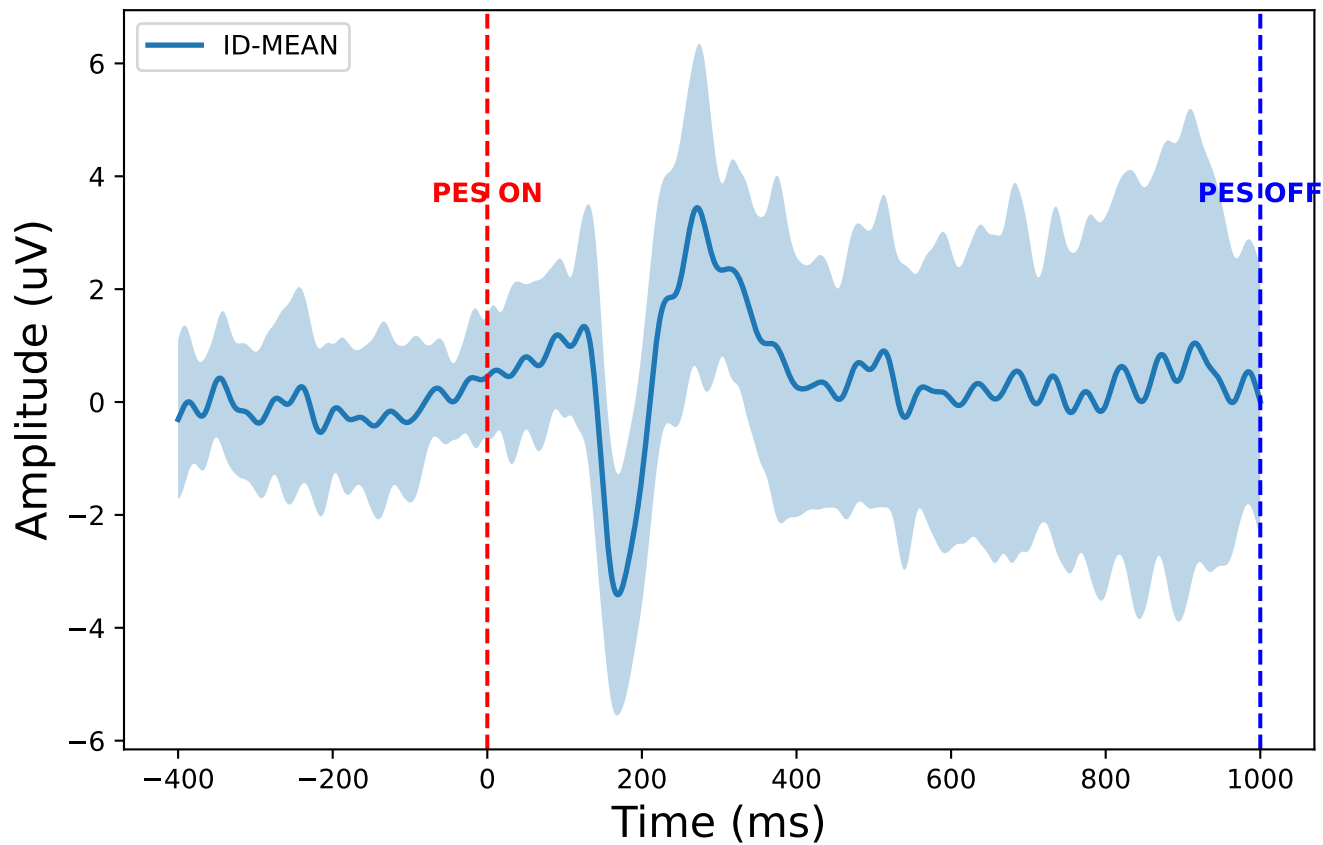

Supplement: Supplementary file 1 [file Supplementary_file_1.zip › Supplementary Material/Data Sheet 6.PDF]

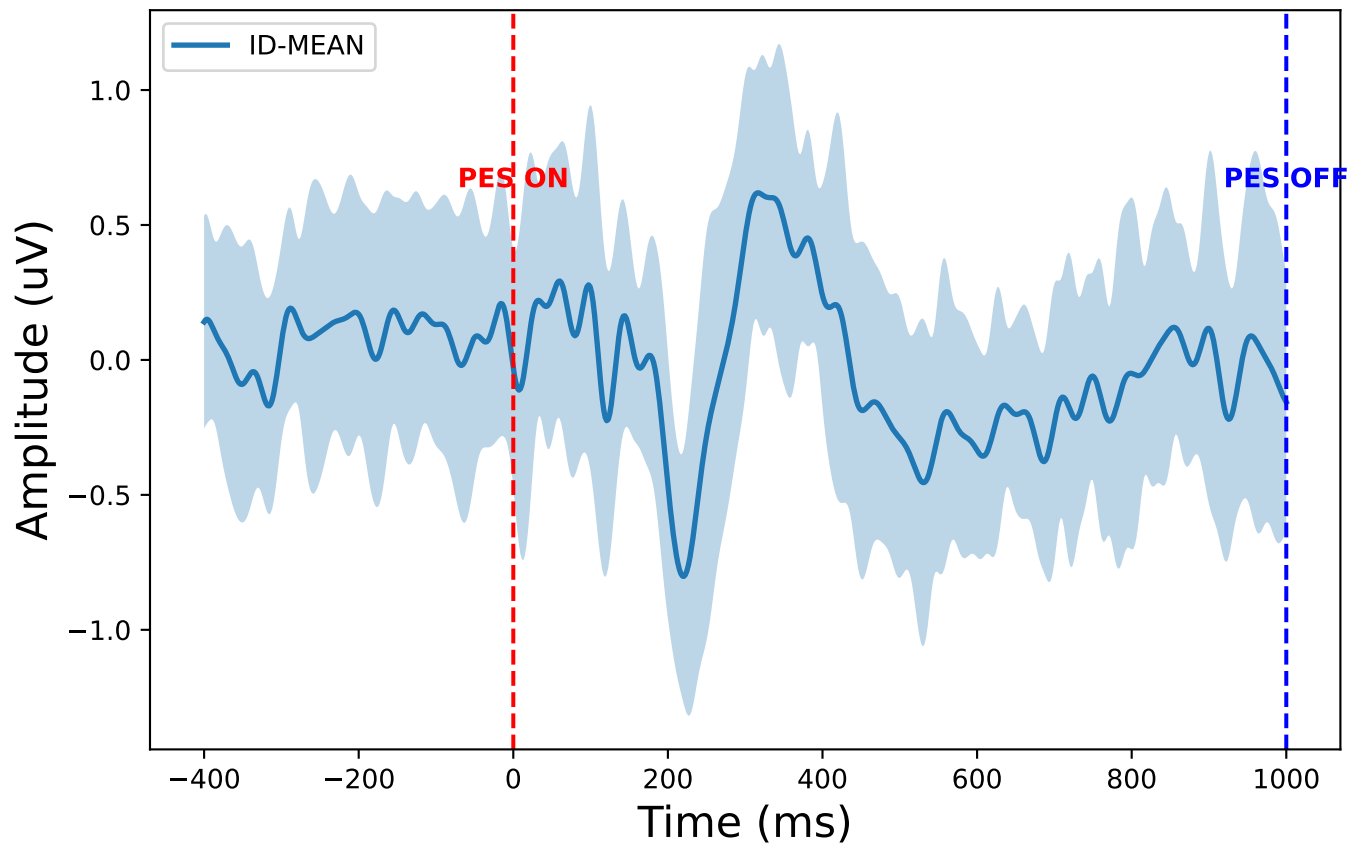

Supplement: Supplementary file 1 [file Supplementary_file_1.zip › Supplementary Material/Data Sheet 7.PDF]

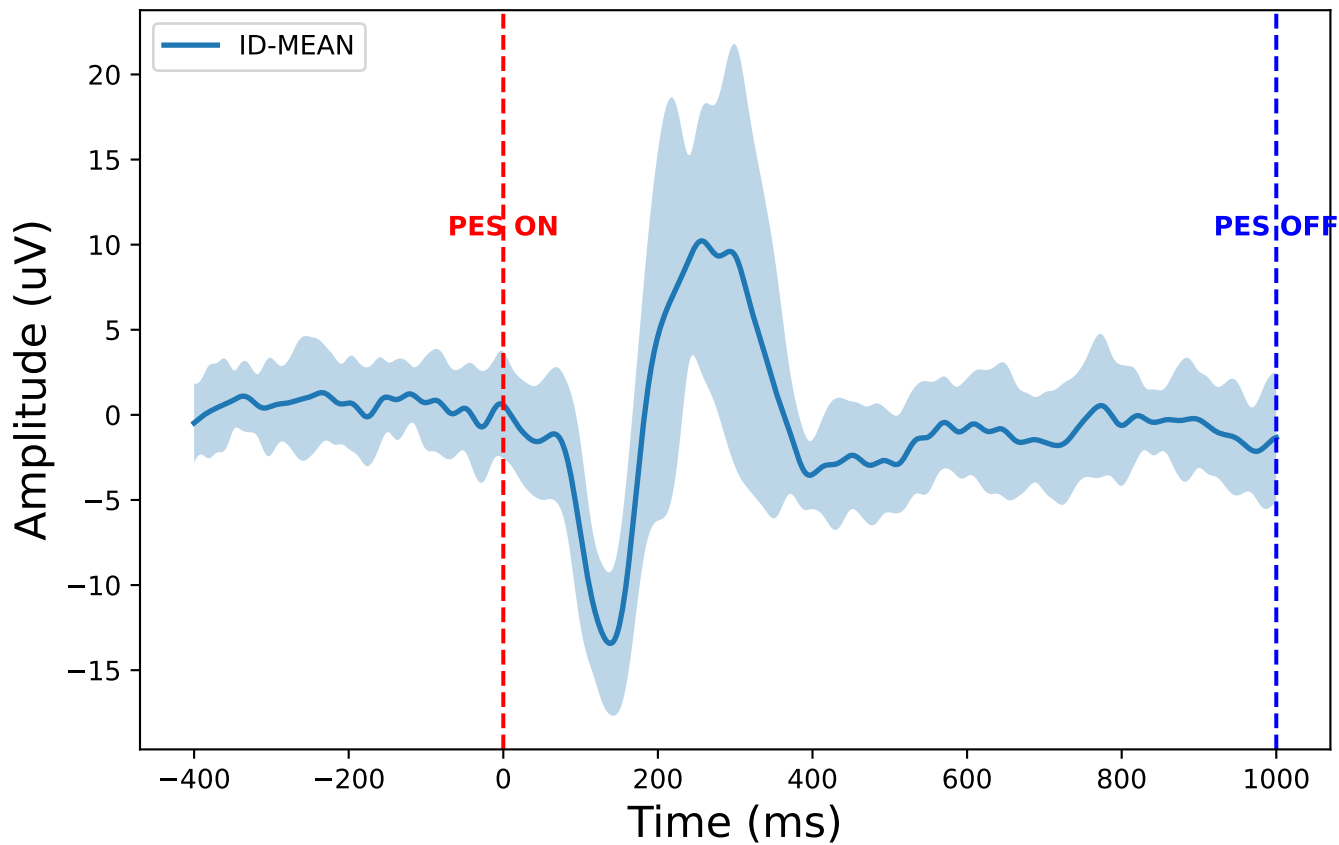

Supplement: Supplementary file 1 [file Supplementary_file_1.zip › Supplementary Material/Data Sheet 8.PDF]

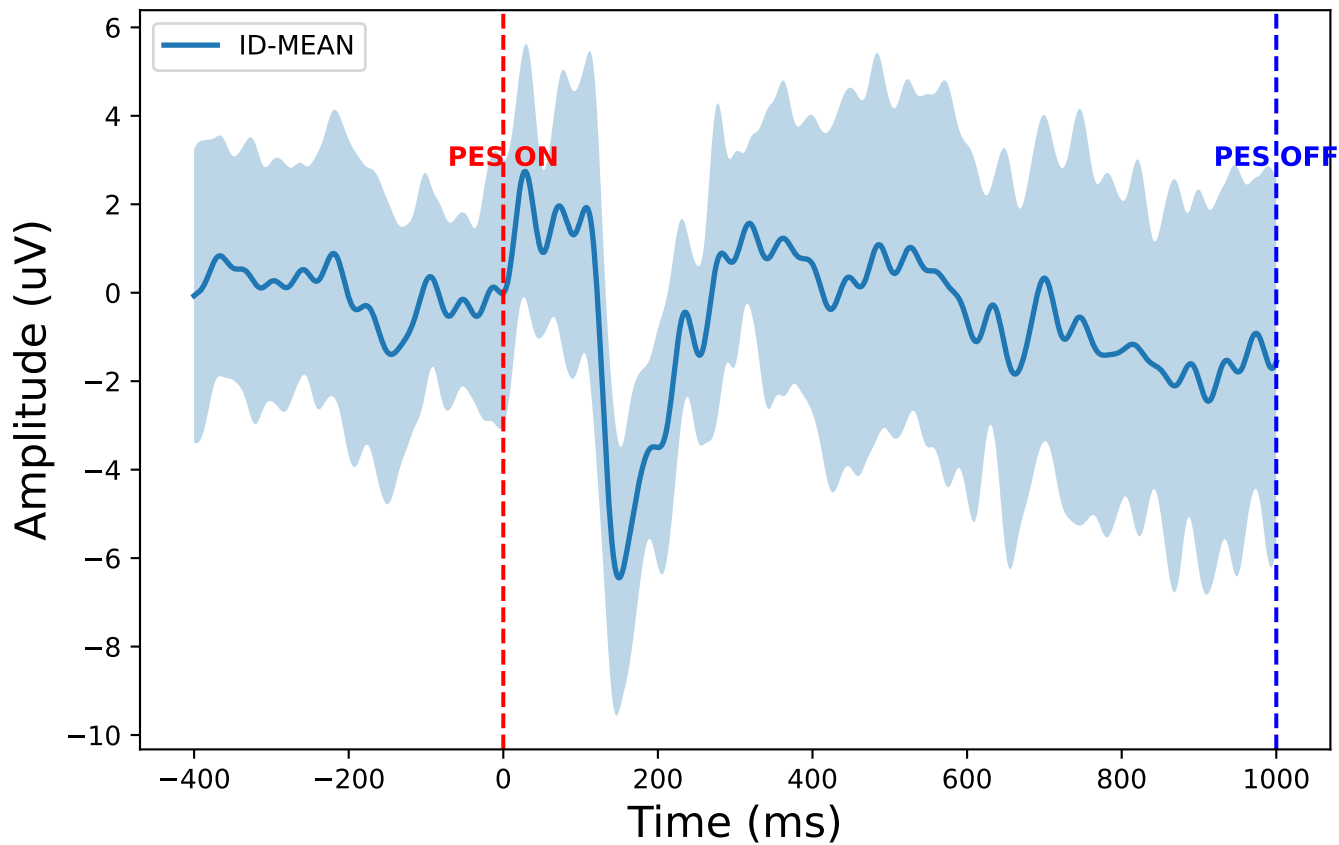

Supplement: Supplementary file 1 [file Supplementary_file_1.zip › Supplementary Material/Data Sheet 9.PDF]
